# A quantitative planar array screen of 440 proteins uncovers novel serum protein biomarkers of idiopathic nephrotic syndrome

**DOI:** 10.1002/mco2.234

**Published:** 2023-05-10

**Authors:** Wei Li, Yan Wang, Binghan Wang, Lin Li, Zhaoyang Peng, Wenqing Xiang, Fei Liu, Haidong Fu, Lidan Hu, Jianhua Mao

**Affiliations:** ^1^ Department of Clinical Laboratory The Children's Hospital, Zhejiang University School of Medicine, National Clinical Research Center for Child Health Hangzhou PR China; ^2^ Department of Nephrology The Children's Hospital, Zhejiang University School of Medicine, National Clinical Research Center for Child Health Hangzhou PR China; ^3^ School of Public Health Zhejiang University School of Medicine Hangzhou PR China

1

Dear Editor,

Idiopathic nephrotic syndrome (INS) is the most frequently diagnosed glomerular disease in pediatric patients. In a nutshell, INS is caused by excessively high glomerular basement membrane permeability, which leads to excessive urine protein excretion.^1^ Based on steroid therapeutic response, INS is categorized into two types, steroid‐sensitive nephrotic syndrome (SSNS) and steroid‐resistant nephrotic syndrome (SRNS). Approximately 80%–90% of pediatric patients respond to steroid treatment within 4 weeks are diagnosed with SSNS, while the remaining 10%–20% are SRNS.^1^ INS might ultimately progress into chronic kidney disease (CKD) and end‐stage renal disease. Early diagnosis might avoid drug toxic side effects and exacerbations. However, there are no biomarkers that can be applied to diagnose INS properly and effectively, especially for the difference between SSNS and SRNS.

As immunosuppression combination with corticosteroids is the mainstay of treatment of INS, mounting evidence indicate that immune activation may be involved in the pathogenic process in the INS.^2^ Whether abnormal level immune proteins are also a hallmark of INS conditions has not been examined in detail. In current study, we established an INS cohort (screening set: 17 cases, validation set: 117 cases) and harvested the serum samples. All serum samples were centrifuged and then screened using the Kiloplex Quantibody protein array platform (GSH‐CAA‐440‐SW, Norcross, Georgia, USA) containing 440 immune‐related proteins (detailed list in Table S1). After normalizing the original data of the protein chip, the differential proteins were screened criteria by corrected *p* value (Benjamini & Hochberg method, ≤0.05) and log_2_Foldchange (expression difference multiple, Foldchange > 1.2). The results of routine analysis of children with INS are shown in Table S2. There were no statistically significant variations in age or gender between the INS group (17 cases) and healthy control group (HC, seven cases).

As shown in Figure 1A, 22 proteins were increased, and 34 proteins were decreased in the INS group compared to HC. The INS group was further subdivided into the SSNS and SRNS group.^3^ Compared to SSNS group, 15 of the 16 proteins increased, while one lowered in the SRNS (Figure 1B). Among these differential proteins, the level of five immune‐related proteins, IL‐12p40, TNF‐β, Adiponectin, TNF‐related apoptosis‐inducing ligand R3 (TRAIL‐R3) and intercellular adhesion molecule 3 (ICAM3), showed mostly observable differences between SRNS and SSNS group. In conclusion, 56 immune‐related proteins were differential expressed on a cohort of patients with INS, and SSNS and SRNS subgroups showed distinct differences in five immune‐related proteins, including IL‐12p40, TNF‐β, Adiponectin, TRAIL‐R3, and ICAM3.

**FIGURE 1:**
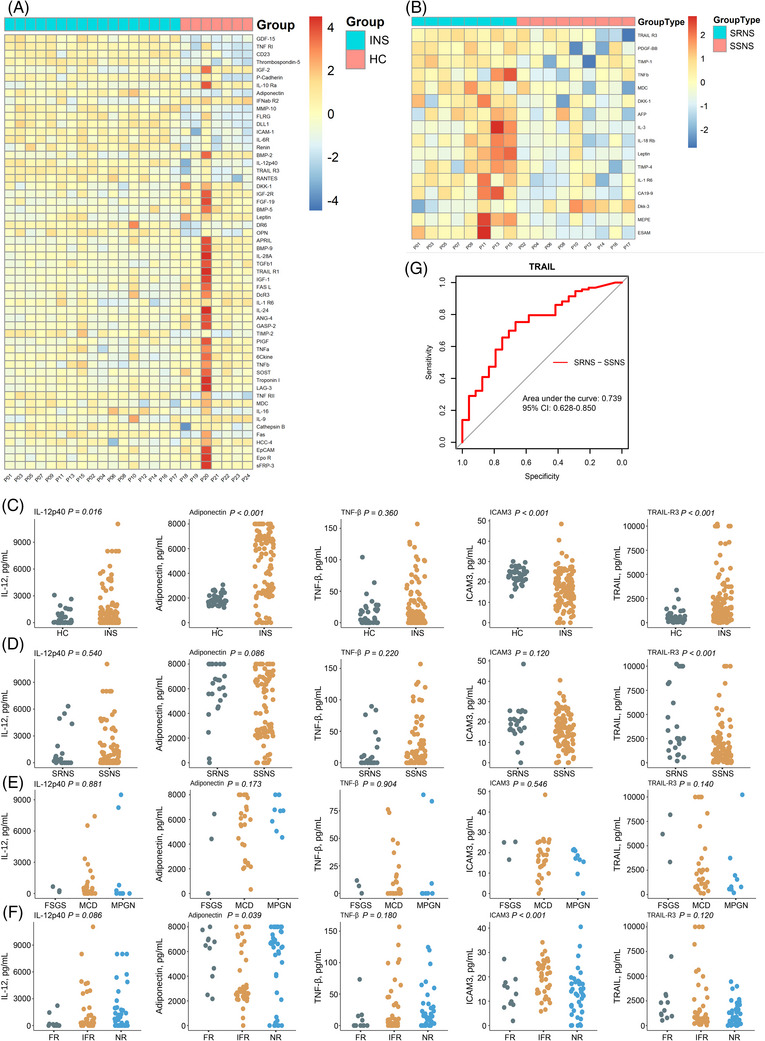
Screening of candidate factors for diagnosis of idiopathic nephrotic syndrome (INS). (A) Heat map of protein chip results of differential proteins in screening set containing 17 INS cases and seven healthy cases. Seventeen INS cases: 11 males and six females, mean age 4.8 ± 2.3 years, and seven control cases: four males, three females, mean age 4.9 ± 1.7 years. The red‐blue color scheme indicates the expression of each of the differential proteins (each cell representing one protein), with red indicating overexpression and blue indicating under expression, compared with the median expression level for that protein. (B) Heat map of protein chip results of differential proteins between SSNS group (*n* = 9) and the SRNS group (*n* = 8). The red–blue color scheme indicates the expression of each of the differential proteins (each cell representing one protein), with red indicating overexpression and blue indicating under expression, compared with the median expression level for that protein. (C) Serum levels of five proteins in validation set containing 117 INS cases and 40 healthy cases. Median levels, pg/mL: IL‐12‐p40: 1,355.43 versus 462.67, *p* = 0.016; adiponectin: 4,878.42 versus 1905.33, *p* < 0.001; TRAIL‐R3: 2216.95 versus 604.90, *p* < 0.001; ICAM3: 16.72 versus 23.15, *p* < 0.001). **(D)** Serum levels of five proteins in SSNS (*n* = 93) children and SSRS (*n* = 24) children (TRAIL‐R3: median levels, pg/mL: TRAIL‐R3: SSNS = 1747.09 versus SRNS = 4,037.64, *p* < 0.001). (E) Serum levels of five proteins among INS children with MCD (*n* = 29), FSGS (*n* = 3), and MPGN (*n* = 9), adiponectin: median levels, pg/mL: FR = 5683.27, IFR = 3740.45, NR = 5227.96; *p* = 0.039. (F) Serum levels of 5 proteins among INS children with different relapse groups (n_FR_ = 11, n_IFR_ = 40, n_NR_ = 42; ICAM3: median levels, pg/mL: FR = 13.25, IFR = 20.10, NR = 12.98; p < 0.001). (G) Receiver‐operating characteristic (ROC) curves for distinguishing SRNS and SSNS using TRAIL‐R3. FSGS, focal segmental glomerulosclerosis; FR, frequent relapse; HC, healthy control; IFR, infrequent relapse; INS, idiopathic nephrotic syndrome; MCD, minimal change disease; MPGN, membranoproliferative glomerulonephritis; NR, non‐relapse; SRNS, steroid‐resistant nephrotic syndrome; SSNS, steroid‐sensitive nephrotic syndrome.

Furthermore, these five proteins were validated using ELISA assays in validation set (INS = 117, control = 40). To assay the level of each protein, serum samples were placed to a microplate precoated with capture antibody, incubated, cleaned, and then captured antibody. Standard curves were used on each ELISA plate to measure the absolute quantification of serum protein indicators. Figure 1C depicted the levels of five proteins in the INS and HC groups. IL‐12‐p40, adiponectin, and TRAIL‐R3 levels were considerably higher, while ICAM3 levels were lower in the INS group, which matched with the above mentioned result of the microarray. However, there was no significant difference in TNF‐β levels. The INS group included 93 SSNS cases and 24 SRNS cases. As shown in Figure 1D, statistically significant difference was only observed in TRAIL‐R3, whereas no significant differences were found in the serum level of IL12‐p40, TNF‐β, adiponectin, and ICAM3. Meanwhile, 41 INS cases identified with pathological examination results after renal biopsy were divided into three groups: minimal change disease (29), membranoproliferative glomerulonephritis (9), and focal segmental glomerulosclerosis (3). No significant difference was found among the three groups (Figure 1E). In summary, The ELISA assay confirmed that the levels of IL‐12‐p40, adiponectin, and TRAIL‐R3 were higher while ICAM3 was lower in the INS group compared to HC group, and that the only statistically significant difference was observed in TRAIL‐R3 when compared SSNS and SRNS groups.

Based on the frequency of onset, INS was divided into group of non‐relapse (NR), non‐frequent relapse (IFR), and frequent relapse (FR).^3^ Except for adiponectin and ICAM3, no differences in the levels of three proteins were found among NR, IFR, and IFR (Figure 1F). The levels of ICAM3 were significantly higher in the IFR group than both in the NR and FR groups. In contrast, adiponectin was significantly lower in the IFR group than both in the NR and FR groups. In brief, the levels of ICAM3 were higher and adiponectin was lower in the IFR group compared to the NR and FR groups.

To analyze the correlation between TRAIL‐R3 and SRNS, the ability of TRAIL‐R3 to rule out SSNS and SRNS was evaluated by receiver‐operating characteristic analysis. The area under the curve for TRAIL‐R3 was 0.739 (95% CI: 0.6275–0.8505). Moreover, a serum TRAIL‐R3 level equal to 2115.921 ng/mL or higher had a sensitivity of 75.3% and a specificity of 66.7% to rule out SRNS and SSNS in children with INS (Figure 1G). To summary, TRAIL‐R3 had a good discrimination ability between SSNS and SRNS.

IL‐12p40 is a chemoattractant for macrophages and increases the migration of bacterially activated dendritic cells. It is linked to pathogenic inflammatory reactions, including silicosis, graft rejection, and asthma. To the best of our knowledge, this is the first scientific way to addressing the fact that IL‐12p40 production is higher in children with INS. Adiponectin, an endocrine substance, was mostly released by adipose tissue, and also a well‐known anti‐inflammatory drug that protected the vasculature, heart, lungs, and intestines. Previous studies also reported that adiponectin was markedly increased in patients with nephrotic syndrome and CKDs in children. Our adiponectin result was consistent with previous reports. ICAM family is a subgroup of the immunoglobulin (Ig) superfamily, with five members (ICAM1‐ICAM5). Numerous studies demonstrated that ICAM3 has a role in immune cell interactions, T lymphocyte activation, acute ischemic stroke and tumourgenesis.^4^ There have never revealed a substantial decrease in ICAM3 children with INS compared to HC group. TRAIL‐R3 is believed to downregulate TRAIL‐induced cytotoxicity by competing for ligand binding with TRAIL‐R1 and TRAIL‐R2. TRAIL may modulate cell survival and proliferation through interaction with two different receptors, TRAIL‐R1 and TRAIL‐R2, and the actions of TRAIL are regulated by three decoy receptors, TRAIL‐R3, TRAIL‐R4, and osteoprotegerin (OPG). The OPG/TRAIL axis has been recently linked to the pathogenesis of renal damage and diabetic nephropathy.^5^ However, no study has ever reported that TRAIL‐R3 may be increased in children diagnosed with INS.

In conclusion, we discovered substantial differences in the expression levels of 56 proteins between children with INS and HC group, as well as significant differences in the expression levels of 16 proteins between the SRNS and SSNS groups. Furthermore, this is the first study to show substantial changes in the expression of IL‐12p40, ICAM3, and TRAIL‐R3 between children with INS and HC group, as well as differences in TRAIL‐R3 expression between the SRNS and SSNS groups. In further study, we will include a larger sample size to verify these newly discovered factors to guide the diagnosis and treatment of INS in children. We will also carry out cross‐sectional study to verify the diagnostic effect of these factors.

## AUTHOR CONTRIBUTIONS

W.L. performed experiments with assistance of Y.W., L.L., and F.L., and P.Z., W.X., and F.H. collected samples. B.W. provided statistical analysis. L.H. and J.M. supervised the project and prepared the manuscript with input from every author. All authors have read and approved the final manuscript.

## CONFLICT OF INTEREST STATEMENT

The authors declare no conflict of interest.

## ETHICS STATEMENT

The study was approved by the Committee on Ethics in the Children's Hospital, Zhejiang University School of Medicine, and written informed consent was obtained from the parents or guardians of all study patients (2020‐IRB‐057). The samples, demographic and clinical information were obtained from patients and healthy subjects who met the inclusion criteria.

## Supporting information

Supporting Informating

## Data Availability

All data are available from the corresponding authors upon request.
