# Supplementary material for: A quantitative planar array screen of 440 proteins uncovers novel serum protein biomarkers of idiopathic nephrotic syndrome
Source: MedComm (2020). 2023 May 10;4(3):e234. doi: 10.1002/mco2.234 (PMC10172734; doi:10.1002/mco2.234)
Supplement: Supplementary file 1 — Supporting Informating [file MCO2-4-e234-s001.docx]

**Title page**

**A quantitative planar array screen of 440 proteins uncovers novel serum protein biomarkers of idiopathic nephrotic syndrome**

Wei Li 1,#, Yan Wang 2,#, Binghan Wang 3, Lin Li 1, Zhaoyang Peng 1, Wenqing Xiang 1, Fei Liu 2, Haidong Fu 2, Lidan Hu 2, *, Jianhua Mao 2, *

*1 Department of Clinical Laboratory, The Children’s Hospital, Zhejiang University School of Medicine,*

*National Clinical Research Center for Child Health, 310052, Hangzhou, PR China*

*2 Department of Nephrology, The Children’s Hospital, Zhejiang University School of Medicine, National Clinical Research Center for Child Health, 310052, Hangzhou, PR China*

*^3^ School of Public Health, Zhejiang University School of Medicine, Hangzhou 310052, PR China*

*^#^ Wei Li and Yan Wang contributed equally to this work.*

# * Correspondence:

Li-dan Hu and Jian-hua Mao, Department of Nephrology, The Children’s Hospital, Zhejiang University School of Medicine, National Clinical Research Center for Child Health, 310052, Hangzhou, PR China

E-mail: [hulidan@zju.edu.cn](mailto:hulidan@zju.edu.cn); jhmao88@zju.edu.cn.

**Materials and methods**

# Study Participants

This study was conducted between January 2019 and September 2020. The diagnosis of INS included edema, 24-h urinary protein excretion of ≥50 mg/kg, morning urinary protein/creatinine of >2 mg, hypoalbuminemia of <25 g/L, and the disease of unknown cause. All children with INS received standard steroid therapy and were divided into two groups: SSNS and SRNS. The SSNS group included patients treated with prednisone [2 mg/(kg d^-1^) or 60 mg/(m d^-1^)] and without proteinuria for ≤ 4 weeks. The SRNS group included patients that failed to achieve remission after 4 weeks of daily prednisone. Furthermore, the relapse group included patients with urinary protein level of ≥ 50 mg/kg, or the urinary protein/creatinine (mg/mg) in morning urine of ≥ 2.0, or the morning urinary protein changed from negative to (+)–(+ + + +) and remained at that level for three consecutive days. The non-relapse group included patients with INS that did not relapse within one year after the first complete remission. The non-frequent relapse group included INS patients that relapsed once within six months or one to three times within one year after the first complete remission. The frequent relapse group included INS patients that relapsed two or more times within half a year or four or more times within one year in the course of the disease. The study was approved by the Committee on Ethics in the Children’s Hospital, Zhejiang University School of Medicine, and written informed consent was obtained from the parents or guardians of all study patients. The samples, demographic and clinical information were obtained from patients and healthy subjects who met the inclusion criteria. Whole blood samples were gathered using plain and EDTA anti-coagulated vaccutainers. Blood in the plain vaccutainers was allowed to clot thoroughly before spinning to obtain serum. Serum was stored at -80 °C forthcoming investigation.

# GSH- protein array platform screen

All serum samples were first centrifuged and then screened using the Kiloplex Quantibody protein array platform purchased from Raybiotech Life (GSH-CAA-440-SW, Norcross, Georgia, USA). The capture antibody for each protein was spotted in quadruplicate onto a glass surface to create the array. Therefore, an n=4 is used for each protein concentration measurement, as these arrays use an 8-point standard curve for each of the 1000 proteins. In brief, all samples were diluted to achieve a total protein concentration within the working range (500 ug/mL – 1 mg/mL). Protein standards and serum samples were incubated on the array for 2 hours to allow the proteins to bind to the antibodies. After washing, a biotinylated antibody cocktail (1000 detection antibodies) was added and left to incubate for 2 hours. Finally, streptavidin-Cy3 was added and left to incubate for 1 hour. After a final wash and dry, the slides were read with a fluorescent scanner. Data was then extracted from the image using a vendor-provided GAL file using compatible microarray analysis software. All data was creatinine normalized before analysis (KGE005, R&D Systems, Minneapolis, Minnesota, USA).

# Validation Studies using ELISA

The identified five biomarkers were validated using ELISA assays, including human IL-12p40 ELISA KIT (4A biotech, China), human TNF-β ELISA KIT (4A biotech, China), Human Adiponectin ELISA KIT (4A biotech, China), human TNF-related apoptosis-inducing ligand R3 (TRAIL-R3) ELISA Kit (CUSABIO, China), human intercellular adhesion molecule 3 (ICAM3) ELISA KIT (LunchangShuo biotech, China). To assay each protein, serum samples were placed to a microplate precoated with capture antibody, incubated, cleaned, and then captured antibody, horseradish peroxidase, and substrate were added. Standard curves were used on each ELISA plate to measure the absolute amounts of serum protein indicators.

**Differential gene pathway analysis**

Protein chip differential gene GO analysis: Fisher exact test was adopted, and the data package was clusterprofiler (R/Bioconductor). The selection standard was that the number of proteins falling on a certain term/GO is ≥ 2, P ≤ 0.05. The term/GO in the plot was arranged in descending order according to the value of count. Enrichment factor was defined as a number of differential genes in a term (count/total number of differential genes) / (total number of genes in the database term / total number of genes in the database).

# Statistical analysis

After the original data of the protein chip was normalized by software, the normalized data was selected for analysis. The analysis method was modeled t-statistics, and the data package was limma from (R/Bioconductor). The differential proteins were screened by corrected P value (BH method) and logFC (expression difference multiple, based on 2). The selection conditions were as follows: (1) logFC > log2 (1.2), and the difference threshold was 1.2; (2) P value after correction ≤0.05.

SPSS 22.0 was used for statistical analysis. Chi-squared test was used to analyze gender differences among groups, and t-test was used for age differences. After the ELISA results were evaluated according to the standard curve, the t-test or Wilcoxon rank sum test were used to compare the two groups, and the analysis of variance or Kruskal-Wallis test was used to compare more than two groups. The difference was statistically significant when P ≤0.05. ROC curve was used to determine the effective area, sensitivity, and specificity of candidate indexes.

**Supplementary tables**

| **Table S1. A novel glass slide-based GSH 440** **immune-related protein microarrays** | | | | | |
| --- | --- | --- | --- | --- | --- |
| GDF-15 | TGFb1 | TSP-1 | PARC | VEGF R1 | IL-13 |
| TNF RI | TRAIL R1 | aFGF | CD40 | CNTF | IGFBP-2 |
| CD23 | IGF-1 | hCGb | BCAM | CEACAM-5 | MCP-4 |
| Thrombospondin-5 | FAS L | RANK | MMP-13 | ADAM12 | IL-17E |
| IGF-2 | DcR3 | IL-17C | RGM-B | EGF | CD58 |
| P-Cadherin | IL-1 R6 | HVEM | IL-17R | ANG-2 | CD155 |
| IL-10 Ra | IL-24 | G-CSF | IL-18 BPa | EG-VEGF | Chemerin |
| Adiponectin | ANG-4 | G-CSF R | MIP-1d | bFGF | SCF R |
| IFNab R2 | GASP-2 | uPA | TREM-1 | Syndecan-3 | TGFb3 |
| MMP-10 | TIMP-2 | Aggrecan | BAFF | HB-EGF | Gas 1 |
| FLRG | PIGF | CD84 | Testican 2 | IL-18 Rb | Fractalkine |
| DLL1 | TNFa | Desmoglein 2 | IL-29 | IL-12p70 | FSH |
| ICAM-1 | 6Ckine | IL-7 | IFNg | JAM-A | TGFb RIII |
| IL-6R | TNFb | IL-11 | LIF | HGF R | Serpin A4 |
| Renin | SOST | IGFBP-1 | BMP-7 | FGF-7 | IGFBP-5 |
| BMP-2 | FAP | VE-Cadherin | Lymphotactin | Leptin R | TARC |
| IL-12p40 | Troponin I | HCC-1 | XEDAR | IGF-1R | IL-15 |
| TRAIL R3 | LAG-3 | IP-10 | Syndecan-1 | LRIG3 | IL-21 |
| RANTES | VCAM-1 | IL-13 R2 | LIGHT | C5a | Tie-1 |
| DKK-1 | TNF RII | IL-2 Rg | AR | IL-4 | CD48 |
| IGF-2R | MDC | IL-5 | SLAM | BTC | GITR |
| FGF-19 | IL-16 | IL-17B | Resistin | IL-8 | FGF-4 |
| BMP-5 | IL-9 | CXCL14 | Tie-2 | PSMA | IL-1 RI |
| Leptin | Cathepsin B | TGFb2 | MMP-3 | Persephin | CA15-3 |
| DR6 | Fas | EMMPRIN | WIF-1 | I-309 | TPO |
| OPN | HCC-4 | SDF-1a | GM-CSF | TACE | E-Cadherin |
| APRIL | EpCAM | IL-13 R1 | Cystatin C | BMPR-II | FGF-9 |
| BMP-9 | Epo R | PDGF Rb | WISP-1 | MCP-3 | Prostasin |
| IL-28A | sFRP-3 | Cadherin-11 | NT-3 | Cadherin-13 | ULBP-2 |
| MIP-1a | MIF | NT-4 | MIP-3a | Siglec-10 | CD229 |
| B2M | ACE-2 | IL-1 RII | CA125 | Clusterin | IL-2 |
| Ck beta 8-1 | Nectin-4 | IL-17B R | MIG | FOLR1 | CD200 |
| NRG1-b1 | B7-1 | BMPR-IB | SDF-1b | Follistatin | DR3 |
| MMP-8 | LDL R | PECAM-1 | LOX-1 | Lipocalin-2 | Contactin-2 |
| Thrombospondin-2 | CD27 | Galectin-3 | BLC | TIMP-1 | IL-31 |
| Pref-1 | SP-D | IL-10 Rb | BDNF | Kallikrein 5 | Cystatin B |
| b-NGF | E-Selectin | DAN | CRP | GRO | EDA-A2 |
| CD6 | GDNF | OPG | GROa | PDGF-AA | Galectin-2 |
| BMP-4 | Nidogen-1 | OSM | Cathepsin L | IL-27 | 4-1BB |
| TRAIL R4 | Albumin | Furin | FABP2 | AFP | MCP-1 |
| IL-1 F9 | BMPR-IA | Angiotensinogen | ICAM-2 | GASP-1 | ADAM8 |
| ST2 | CD40L | Osteoactivin | IL-1 F7 | Notch-1 | ANG-1 |
| SIGIRR | IL-1a | IL-1 R5 | Fcg RIIBC | IL-23 | FGF-6 |
| ESAM | PF4 | Eotaxin-2 | NCAM-1 | CHI3L1 | TLR2 |
| MPIF-1 | IL-2 Ra | Transferrin | TRAIL | NGF R | CD163 |
| I-TAC | Fetuin A | RBP4 | ICAM-3 | PAI-1 | gp130 |
| Dtk | CA9 | TRAIL R2 | GH | IGFBP-6 | Pentraxin 3 |
| Axl | CTLA4 | Dkk-3 | MMP-2 | CRTAM | CTACK |
| Ferritin | IL-1 F8 | CEA | Thyroglobulin | Pepsinogen I | TSH |
| Cystatin A | TWEAK | VEGF | ErbB4 | IL-18 | CD99 |
| Adipsin | GITR L | MEPE | Layilin | Follistatin-like 1 | IL-1b |
| IL-3 | ULBP-1 | CD30 | IL-15 R | L1CAM-2 | Dkk-4 |
| bIG-H3 | CCL28 | VEGF R3 | BCMA | Galectin-9 | TIM-1 |
| ICOS | MIP-1b | GCP-2 | ADAM9 | LIMPII | Cripto-1 |
| IL-21R | HAI-2 | ErbB2 | IL-6 | Mer | NOV |
| IL-34 | DPPIV | AMICA | SCF | L-Selectin | Thrombomodulin |
| TECK | DNAM-1 | Eotaxin | MIP-3b | LAP(TGFb1) | TF |
| PSA-free | HGF | MMP-9 | TIMP-4 | IL-1 R3 | FGF-21 |
| TRANCE | Siglec-9 | IL-10 | Marapsin | Syndecan-4 | VEGF-C |
| Legumain | CA19-9 | NSE | Galectin-1 | MCSF R | NrCAM |
| EGF R | Insulin | AgRP | B7-H3 | Angiogenin | Prolactin |
| IGFBP-4 | Eotaxin-3 | IL-2 Rb | MMP-1 | Granulysin | Flt-3L |
| NAP-2 | TIM-3 | ANGPTL4 | MICA | Neprilysin | IL-33 |
| IL-32 alpha | PDGF-BB | Midkine | PDGF-AB | CEACAM-1 | Kallikrein 14 |
| MICB | Endoglin | uPAR | IL-1 F5 | MBL | TFPI |
| MSP | Cystatin E M | Angiostatin | CF XIV | IL-1ra | IL-5 Ra |
| Cadherin-4 | ADAMTS13 | JAM-B | Shh-N | MCSF | VEGF-D |
| VEGF R2 | S100A8 | IL-1 F6 | CD97 | IGFBP-3 | TACI |
| Procalcitonin | RAGE | IL-17F | TLR4 | Trappin-2 | Activin A |
| 2B4 | ENA-78 | Decorin | TSLP | B7-H1 | IL-20 |
| MMP-7 | MCP-2 | ErbB3 | CXCL16 | LYVE-1 | Siglec-7 |
| Galectin-7 | ALCAM | TGFa | Cathepsin S | Periostin | PD-1 |
| CD14 | PGRP-S | ANGPTL3 | Siglec-5 | ROBO3 | IL-1 F10 |
| IL-17 | LRP-6 |  |  |  |  |

More detailed information could be found elsewhere (<https://www.raybiotech.com/?match=all&subcats=Y&pcode_from_q=Y&pshort=Y&pfull=Y&pname=Y&pkeywords=Y&search_performed=Y&match=all&cat_search=Y&cid=0&hint_q=GSH-CAA-440&dispatch=products.search>)

**Table S2. Routine analysis of children with INS.**

| **Groups** | Random urine protein | Blood creatinine | Random urine creatinine |
| --- | --- | --- | --- |
| SSNS | +- → ++++ | 15 → 69 | 557 → 19687 |
| SRNS | + → ++++ | 14 → 70 | 621 → 23449 |

All routine analysis results were obtained from retrospective data.

**SSNS**: Steroid-sensitive nephrotic syndrome, **SRNS**: Steroid-resistant nephrotic syndrome.
